# Supplementary material for: A qualitative study of behavioral and social drivers of COVID-19 vaccine confidence and uptake among unvaccinated Americans in the US April-May 2021
Source: PLoS One. 2023 Feb 10;18(2):e0281497. doi: 10.1371/journal.pone.0281497 (PMC9917274; doi:10.1371/journal.pone.0281497)
Supplement: S1 File — (DOCX) [file pone.0281497.s001.docx]

**S1. Screening Questionnaire**

**Introduction**

**INTRO [DISP]**

Thank you for participating in this KnowledgePanel® survey; it should take less than 5 minutes to complete. We have a few questions about your daily life and interests. We’re interested in your opinions, so please respond to questions based on what you think — there are no right or wrong answers.

As with all KnowledgePanel® surveys, responding to this survey, or to any individual question on the survey, is completely voluntary. Your responses remain anonymous and will be used for research analyses only.

If you have questions about your rights as a participant in this survey, or if you are dissatisfied at any time with any aspect of the survey, you may contact KnowledgePanel® Member Support at 800-782-6899.

**Section 1**

Base: All respondents

**S1. [S]**

In general, would you say your physical health is…

1. Poor

2. Fair

3. Good

4. Very good

5. Excellent

Base: All respondents

**S2. [S, prompt once]**

Have you received at least one dose of a COVID-19 vaccine?

1. Yes **[THANK and TERMINATE]**

2. No

Base: S2=2 (Have not received COVID-19 vaccine)

**S2a. [S, prompt once]**

How likely are you to get a COVID-19 vaccine?

1. Definitely will not

2. Probably will not

3. Not sure

4. Probably will **[THANK and TERMINATE]**

5. Definitely will **[THANK and TERMINATE]**

**Section 2 - Recruit**

Base: S2a=1, 2, 3

**S3 [S, prompt once]**

Thanks for answering our questions! You are one of a select group who may be invited to participate in a follow-up activity that we call an “online focus group.” If selected, we will ask you to participate in online interactive activities where you can express your opinions on your COVID-19 experiences.

An Ipsos professional will lead the online focus group. There will be other people from across the country participating. You will get 75,000 points if you are selected and agree to participate. In order to receive the bonus points, you need to actively participate in the session.

Would you be willing to participate in an online focus group on this topic?

1. Yes
2. No **[THANK and TERMINATE]**

Base: S3=1 (Yes)

**S4 [S, prompt once]**

Are you available to participate in the online focus group at the following date and time?

**[INSERT Date]** at **[INSERT Local_Time]**

1. Yes

2. No **[THANK and TERMINATE]**

Base: S4=1 (Yes)

**Consent.** **[S]**

**PURPOSE OF THE STUDY**

You are one of a select group who may be invited to participate in a follow-up activity that we call an “online focus group.” Please note, for the purposes of this focus group, you will need to connect using a computer or laptop with a working microphone and camera. If selected, we will ask you to participate in an online group meeting where you can express your opinions on these and other issues.

**WHAT WILL HAPPEN DURING THE STUDY**

This online focus group will be led by an Ipsos professional and conducted via Zoom, for which we will send you a link. In order for you to participate, we need to pass along your name, email address, and phone number to an Ipsos partner company called Focuscope. Focuscope will call you before the online focus group to perform a technology check. Your information would be used for this study only. At no time will any attempt be made to sell you anything nor will you receive any sales calls or emails once you participate in this focus group.

**LENGTH OF THE STUDY AND NUMBER OF PARTICIPANTS EXPECTED TO TAKE PART**

Around 5-10 participants above age 18 are expected to participate in the online focus group. The online focus group will last about 60 minutes.

**POSSIBLE RISKS OF THE STUDY**

There are no known risks with this study. Answering some of the questions may make you uncomfortable.

**POSSIBLE BENEFITS OF THE STUDY**

This study will provide no direct benefit to you.

**PAYMENT FOR BEING IN THE STUDY**

75,000 bonus points (worth $75) will be offered for participating in this online focus group.

**ALTERNATIVES TO PARTICIPATION**

Your only other choice is not to take part in this research study.

1. Yes, I am willing to participate

2. No, I am not willing to participate **[THANK and TERMINATE]**

Base: Consent=1 (Yes)

**Qend.** **[DISP]**

Thank you for agreeing to participate! If you are selected, we will contact you soon with further details regarding the online focus group. We will send all further information for this project with the subject of “Your Health Today,” so keep an eye out for it!

**END OF INTERVIEW**
