# Supplementary material for: A qualitative study of behavioral and social drivers of COVID-19 vaccine confidence and uptake among unvaccinated Americans in the US April-May 2021
Source: PLoS One. 2023 Feb 10;18(2):e0281497. doi: 10.1371/journal.pone.0281497 (PMC9917274; doi:10.1371/journal.pone.0281497)
Supplement: S2 File — (DOCX) [file pone.0281497.s002.docx]

**S2 File. Discussion Questions**

**Background**

This document provides sample questions and a note-taking template that can be used when conducting key informant interviews (KIIs), listening sessions, or focus group discussions to assess COVID-19 vaccine access, demand, and confidence. These questions may be used by a polling company or by state and local health department staff involved in various aspects of COVID-19 vaccine rollout planning; relevant staff may include immunization program managers, epidemiologists, immunization information system (IIS) staff, health educators, or others.

Use the questions below to facilitate the main discussion. Adapt questions and add more probes as needed to elicit detailed information.

**A. General Introduction –**

1. To start off, it would be helpful to understand how COVID-19 has affected you or your community through the course of this pandemic.

*Probes:*

1. Activity Impacts
   - 1. Lockdowns
     2. Work impacts
        1. Remote work
        2. Closures
     3. Service disruptions (childcare, restaurants and bars, outside recreation, socializing restrictions)
     4. Lifestyle disruptions (schools, libraries, travel, team sports)
     5. Organizational disruptions (faith, community engagement)
2. Health Effects
   1. Postponing care (for yourself, your family, community members)
   2. Other health impact (availability of tests, pharmacy, etc.)
   3. Morale or psychological effects of COVID-19 (anxiety, sadness, etc.)

**B. COVID-19 Vaccine Attitudes in the Community –**

1. What do you think about COVID-19 vaccines?
   1. Comments might vary based on
      1. Vaccine type or product
2. What do people in your daily life think about COVID-19 vaccines?
   1. What are some things you have heard from your community about the vaccine(s)?
   2. Vaccine Distinctions (old, new)
      1. Do not probe unless raised by participants – if yes, then explore further
3. Do you think most people in your community would be willing get a vaccine once it becomes available to them?
   1. Why?
   2. Why not?

**C. Barriers and Enablers to COVID-19 Vaccination in the Community**

1. How do you think the introduction of COVID-19 vaccines will affect you or your community?

*Probes:*

1. Lifting of social distancing restrictions
2. Restoration of social and community activities, access to institutions/organizations
3. Other environments (restaurants, schools, faith-based organizations, etc.)
4. What are the main reasons people in your community would want to get vaccinated?

*Probes:*

- 1. Life going back to normal
  2. Community (herd) immunity
  3. Work requirements

1. What are the main reasons people in your community may not want to get vaccinated?

*Probes:*

- 1. Information
  2. Misinformation
  3. Attitudes toward vaccines and vaccination
  4. Fear of side effects
  5. Trust in government, medical system, healthcare workers

1. How easy do you think it is for people who are eligible in your community to get a COVID-19 vaccine if they wanted to?

*Probes:*

- 1. Issues related to access to health facilities/clinics
  2. Work conflict
  3. Household dynamic

1. Are there any key barriers that people in your community are likely to face if they went out to get a COVID-19 vaccine?
   1. Barriers that currently exist
   2. Perceived barriers in the future

**D. Information Sources, Rumors and Sentiment in the Community**

1. There is a lot of information about the COVID-19 vaccines out right now. What have you heard about the COVID-19 vaccines from the sources you trust?
   1. Probe: gather list of sources
2. How about from sources you don’t trust?
   1. Probe: gather list of sources
3. What do you do when you hear information about COVID-19 vaccines that you aren’t sure is accurate?
   1. Possible probes include
      1. This sounds suspicious to me
      2. Too good to be true
      3. Information makes you concerned about the vaccine
   2. Where do you get this information?
4. Do you share this information (such as via social media)? If so, when and why do you share this information?
   1. What information is important for you to share?
   2. What prompts you to share this information?
5. How do you share information?
   1. List of modes:
      1. Email
      2. Text
      3. Direct message platforms (e.g., WhatsApp, Signal)
      4. Social media (e.g., Twitter, Facebook, Instagram, Gab)

**E. Strategies to Improve Vaccine Confidence in the Community**

1. What organizations and institutions in your life are important to you?

*Probes:*

- 1. Faith-based organization
  2. School
  3. Local park/recreational department
  4. Library
  5. Community center
  6. Arts and cultural centers
  7. Social media
  8. Friends (and friend groups – parent groups, kids sport teams)
  9. Local government
  10. Book club
  11. Political group

1. How do you think organizations and/or institutions in your community/life can contribute to more people in your community having confidence and access to a COVID-19 vaccine?

*Probes:*

- 1. Faith-based organization
  2. School
  3. Local park/recreational department
  4. Library
  5. Community center
  6. Arts and cultural centers
  7. Social media
  8. Friends (and friend groups – parent groups, kids sport teams)
  9. Local government
  10. Book club
  11. Political group

1. How do you think your healthcare provider/other healthcare providers in your community or daily life can play a role in encouraging or convincing people to take the vaccine?
   1. Probe on who are important health related influencers in their daily lives
      1. Primary care providers
      2. Health departments (municipal, state, federal)
      3. Others
2. Are there specific individuals in your community (such as influencers) that can play a role in encouraging or convincing people?
